# Effects of growth technique on the microstructure of *CuInSe*_2_ ternary semiconductor compound

**DOI:** 10.1016/j.heliyon.2020.e03196

**Published:** 2020-01-21

**Authors:** M. Mobarak, M.A. Zied, Massaud Mostafa, M. Ashari

**Affiliations:** a Physics Department, College of Science, Jouf University, P.O. Box: 2014, Sakaka, Saudi Arabia; b Physics Department, Faculty of Science, South Valley University, Qena 83523, Egypt

**Keywords:** Condensed matter physics, Materials science, Chalcopyrite, Microstructure, Grain size, Microstrain, Stochiometry

## Abstract

X-ray diffraction (XRD) and Energy-dispersive X-ray fluorescence spectrometer (EDXRF) are employed to investigate the microstructure of bulk $CuInSe_{2}$ specimens grown through the Bridgman technique and traveling heater process, respectively. We investigate the lattice parameters, grain sizes, and microstrains of the two grown samples. For a crystal grown by the vertical Bridgeman method, the vacancy $V_{Cu}$ serves as an acceptor, resulting in p-type conduction, whereas the vacancy $V_{Se}$ expected to serve as a donor, occurring in n-type conduction for the crystal grown via the traveling heater technique. The concentration of crystal grown via the VBM is determined to be p-type, whereas the concentration of that grown via the THM is n-type. Concerning $CuInSe_{2}$ crystal, the determined crystallite sizes obtained to be 165 and 182 nm for the VBM and THM, respectively.

## 1 Introduction

$CuInX_{2}$ (x = Se, Te or S) is one of the I-III–VI2-type compound semiconductors together with a chalcopyrite structure. Its bandgap is in the energy domain for optimal solar conversion and its absorption coefficient is very high ($5\times 10^{-5}$ cm^−1^ near the bandgap) [1]. The threefold chalcopyrite semiconductor $CuInX_{2}$ has got considerable concern for photovoltaic applications [2]. Latterly, $CuInX_{2}$-based heterojunction interface solar cells were denoted to secure an efficiency of 14.1% [3]. The vertical Bridgman technique (VBTs) was found to be the widest method applied in growing CuInS2 single crystals [4], [5], [6] and the traveling heater method (THM) [7]. As it is recognized from the $CuSe_{2}-In_{2}Se_{3}$ phase diagram [8], the stoichiometric melt-grown $CuInX_{2}$ crystal undergoes a solid-state variation at 810 °C, transforming the sphalerite compound CuInX2 into the chalcopyrite compound. When the growth process was performed through the solution growth method below 810 °C, high-quality bulk expected to be obtained with the chalcopyrite compound because the crystals grow without phase mutation.

In this study, we investigated the effect of growth methods on the structural properties of $CuInX_{2}$ grown using the vertical Bridgman method (VBM) and (THM). In the THM technique, we used indium (In) as a solvent. A temperature of 751 °C was preferred, such that the territory satiated with stoichiometric $CuInX_{2}$ solute. Many different research studies similar to this work carried out [9], [10], [11], [12], [13], [14] without giving details about the effects of growth technique on the microstructure. Besides, the atomic positions were specified from X-ray powder diffraction by the Rietveld refinement method.

## 2 Experimental procedures

### 2.1 Crystal growth: Vertical Bridgman Techniques (VBTs)

$CuInX_{2}$ crystals were grown by VBTs in an evacuated quartz ampoule. A quartz tube was first cleaned with distilled water and acetone to remove organic impurities and then with a mixture of Hf and distilled water in the ratio of 3:1 for five h to remove any metallic impurities. After the ampoule was rinsed with hot and cold purified water several times, the ampoule baked at 1000 °C for 5-6 h under vacuum for out-gassing and was coated with carbon by methane pyrolysis at 1000 °C. It was then ready for material growth. A composition of the advised amount of elementary copper, selenium, and indium, corresponding to the formation of stoichiometric $CuInX_{2}$, was placed into a carbon-covered quartz ampoule of 9.1-mm diameter and 2-mm guard thickness. The weight of the examined mixture was around 11-15 g. The impurity of CuIn was 6N grade, and that of Se was 5N grade. After evacuation to a pressure of $3.1\times 10^{-6}$ Torr, the length of the sealed off ampoule was around 8.1 cm. The ampoule is then placed in a standing oven and heated up to 1101 °C. To control the high Se vapor pressure and the exothermic reaction between In and Se, a watchfulness when heating the ingot casting considered. The mixture was gradually heated (with the rate of 0.5 to 1 °C/min) over a temperature interval of 200 to 250 °C to reduce the risk of cracking the ampoule. The temperature value was conserved at 1101 °C for 48 h for complete reaction and homogenization of the charge. The crystal growths performed by lowering the ampoule at a speed of 1.33 mm/h for 17 days.

### 2.2 Crystal growth: Traveling Heater Method (THM)

About the THM, a specific carbon-coated quartz ampoule with a diameter of 10 mm used for the growth of $CuInSe_{2}$. 1.0 g ingots of Indium placed in the ampoule as a zone solvent and the $CuInSe_{2}$ polycrystalline ingot as a feed material. After evacuation to a pressure of $3.1\times 10^{-6}$ Torr, the length of the sealed off ampoule was about 6 cm and then placed in a standing THM oven possessing three-loop heaters (main, up-sub- and low-sub-heaters). The measured temperature profile of the stove was along the axis and without the ampoule. The highest temperature was 781 °C, and the slope at 750 °C is around 40 degrees centigrade per centimeter. The wideness of the zone on the axis where the temperature was higher than 750 °C was around 1.51 cm. Crystal growth performed by lowering the ampoule at a rate of 0.4 cm/day for ten days.

### 2.3 Energy-dispersive X-ray fluorescence spectrometer (EDXRF)

The quantitative analysis of our samples investigated by using the (JSX-3222) Element Analyzer together with Energy Dispersive X-ray Fluorescence spectrometer (JEOL, Japan) [13]. The quantitative analysis made by comparison with known standards. The micro-analysis indicated that VBM prepares the produced crystals, and THM Method was stoichiometrically compound corresponding to $CuInSe_{2}$ semiconductor.

### 2.4 X-ray diffraction (XRD)

XRD of the grown $CuInSe_{2}$ system performed using an automatic X-ray diffractometer (Philips PW1710 Diffractometer). The pattern runs with Mo as the target and a graphite monochromator (λ=0.71069 Å) supplying the generator with a power of 50 kW at 30 mA. The measurements carried out at room temperature with a scanning speed of 3.76 deg/min. Measurements were carried out by the step scanning method with a stepping angle of 0.25° in the range of 4–100°. The recorded X-ray diffraction used for calculating the grain size and microstrain from the profile breadth. The profile width was deliberate using computer software as the full-width at half-maximum (FWHM) after automated background removal and $k_{\alpha 2}$ stripping (stripping ratio $k_{\alpha 2}/k_{\alpha 1}$).

### 2.5 Thermoelectric Power Test (TEP)

TEP Hot prob method used to identify the conductivity type of our grown samples. The sign of thermo-voltage between both ends of our sample found to be positive, and this means that the major carriers are holes. Hence, the crystal grown by VBM is a P-type semiconductor, whereas the crystal grown by THM found to be an n-type semiconductor.

## 3 Results and discussion

### 3.1 Elemental composition

To study the elemental composition of the two-grown crystals, EDXRF used. The results indicated that for crystal growth by VBM, the mean values of Cu = 24.374%, In = 25.138%, and Se = 50.488%, in this part found to be in good agreement with the composition based on the ideal stoichiometry, where a redundant of Se and a slim deficit of Cu observed. The redundant of indium and selenium refers to the stoichiometric composition. On the other side, in THM the mean values for Cu = 25.853%, In = 25.105%, and Se = 49.042%, in this part were found to be in good agreement with the composition based on the ideal stoichiometry, where a small redundant of Cu and slight deficit of Se observed. In general, these results are in good agreement with the compositions of the $CuInSe_{2}$ grown by other methods. Electron microprobe analysis of the uppermost cluster revealed the material to be very nearly stoichiometric $CuInSe_{2}$ with an atomic composition of Cu: 24.7±0.2, In: 25.7±0.2 and Se: 49.7±0.5. In contrast, a crystal from the lower cluster showed a large deficiency of copper with a composition of Cu: 22.7±0.2, In: 26.7±0.2 and Se: 51.7±0.3 [15].

Electron microprobe analysis of $CuInSe_{2}$ with a 1 μm beam diameter used to investigate the composition of one plate on the grown surface. Ten measurements indicated an atomic composition of Cu: 24.8% (with 0.3 estimated error), In: 25.4% (with 0.3 estimated error), and Se: 48.8% (with 0.6 estimated error), and these values were in good agreement with the values taken by Ciszek with a slight difference of 1.0% (with 0.01 estimated error) [16].

### 3.2 Conductivity type

The deviations by Ref. [17] from the ideal formula x:y:z = 1:1:2 can be reported by two important variables, the non-molecularity Δm and non-stoichiometry Δz. These two specified as

(1) Δm=([Cu]/[In])−1

(2) Δz=(2[Se]/[Cu]+3[In])−1

If the sign of Δm and Δz is recognized, it will be possible to make predictions regarding the type of the conductivity and probable electrically active point deficiency [18]. If Δm<0, Δz>0, the anticipated defect is a $V_{Cu}$ deputing as an acceptor (p-type), and if Δm>0, Δz<0, the vacancy $V_{Se}$ deputing as a donor is expected (n-type). The sign of Δm>0, Δz <0 for the two crystals is listed in Table 1. It can easily figure out that for the VBM crystal and Δm <0 and Δz >0, the anticipated defect is a $V_{Cu}$ acting as an acceptor. For the THM crystal with Δm >0 and Δz <0, the vacancy $V_{Se}$ acting as a donor is expected. The concentration of crystals grown via VBM is determined as a p-type, whereas that grown via THM is an n-type. This result is in agreement with the TEP probe result.

**Table 1:** Microstructural parameters of the VBM and THM crystal investigated from X-ray line broadening by using Scherrer, Williamson–Hall, and Warren Averbach equations, respectively.

G. tech.	Type		Scherrer	Williamson-Hall			Warren Averbach				
	Δm	Δz	<*L*>_*vol*_	*L*_*vol*_	*D*_*vol*_	M.s	<*L*>_*area*_	<*L*>_*vol*_	*D*_*area*_	*D*_*vol*_	M.s
VBM	<0	>0	165	125	167	0.0042	92	129	138	172	0.0038
THM	>0	<0	182	133	177	0.0055	102	135	153	180	0.0044

### 3.3 Lattice parameter

**Figure 1:**
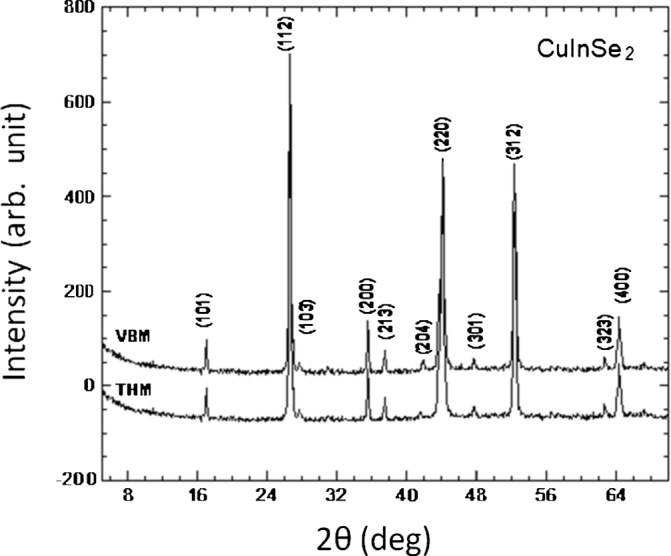
The X-ray diffraction pattern of VBM and THM crystals.

Fig. 1 presents X-ray powder diffractograms of the two grown $CuInSe_{2}$ specimens. The strong Bragg peaks of the as-grown $CuInSe_{2}$ system indicate crystallinity. Besides, the Bragg peak of the two crystals is the same, with only a small displacement in the position of this peak, and varies in intensity and FWHM. The main peaks for the two crystals are (101), (112), (103), (200), (213), (220), (301), (303), (312), (323), and (400), which refer to the tetragonal $CuInSe_{2}$ crystal. The lattice parameter a and c were obtained substantially free from experimental error. They obtained by plotting the apparent value of a and c, calculated from each reflection plane, against corresponding values of:

(3) $$f(\theta )=\frac{1}{2}(cos^{2}\theta /sin\theta )+(cos^{2}\theta /\theta )$$

and extrapolating to f(θ)=0 [19], [20], [21]. The results indicate that the mean values of the lattice parameters of the crystal prepared by the Bridgman method are a = 5.7823 Å, c = 11.6192 Å and c/a=2.00855, whereas those of the crystal grown by the THM crystal are a = 5.787 Å, c = 11.574 Å and c/a=2. These values are in good agreement with the previous studies. Igarashi [1] reported that the growth of tetragonal $CuInSe_{2}$ (a = 5.782, c = 11.622, and c/a=2.01) occurs in such a way that 3-crystallographic axes of $CuInSe_{2}$ coincide.

With those of the zinc-blende-type substrate, there are five credible cases of growth in which the $CuInSe_{2}$ crystal grows on the (001) zinc-blende-type substrate. Afterward cooling process to room temperature, the materials were recognized via X-ray diffraction measurements as a single-phase with lattice constants a = 5.783 and c = 11.618 with c/a=2.009 [6]. The crystals grown by the two methods, VBM and THM, were investigated by the back-reflection Laue method and the X-ray diffractometry. Specimens from different parts of the ingot were examined to be of a chalcopyrite composition with lattice parameters a = 5.7824 and c = 11.6192 with c/a=2.009 [6].

### 3.4 Structure refinements

**Figure 2:**
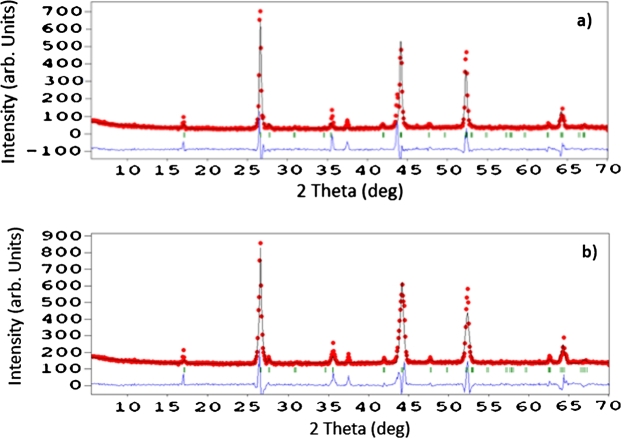
Observed, calculated, and different profiles for the final Rietveld refinement for sample pattern of a) THM and b) VBM crystals.

In this part, we have tried to relate the structural parameters to the vertical Bridgman method (VBM) and (THM) method of the $CuInSe_{2}$ preparation technique. The atomic positions were determined from X-ray powder diffraction by the Rietveld refinement method, and the Se site found to be associated with the Cu content. Least-squares structure refinements assumed with the FULLPROF Rietveld type program [22]. The refinement was initiated by assuming a = b = 5.7955 Å, c = 11.6464 Å, x Se = 0.22705. The first background parameter initially estimated by WINPLOTR-2006 inspection of the observed diffraction patterns. Fig. 1 shows a plot of the observed, calculated, and different profiles for the final Rietveld refinement for sample pattern of prepared by THM method (Fig. 2a) and by VBM method (Fig. 2b). The next quantities used to measure the agreement between the observations and the model during the progression of the Rietveld refinements: the pattern R factor (Rp), the weighted pattern R factor (Rwp), the expected R factor (Rexp), the Bragg intensities R factor (RB), the structure amplitudes R factor (RF), and the (goodness of fit) defined by the ratio GF = Rwp/Rexp. The quantity minimized in a Rietveld refinement is the weighted profile R-value, Rwp, but its numerical value may be misleading. Thus, it is not the value of the minimum reached in the weighted profile R factor, but the structure parameter set (RB) and (RF) obtained from the minimum value, that is, the more significant. Table 2 shows the agreement indices obtained after Rietveld refinements. The final Rietveld refinement parameters obtained, as shown in Table 3.

**Table 2:** Agreement indices after Rietveld refinement.

	Rp	Rwp	Rexp	RB	RF	GF
THM crystals	50.0	40.9	32.35	60.15	35.24	1.26
VBM crystals	46.4	32.4	32.87	25.99	24.79	0.98

**Table 3:** Final Rietveld refinement parameters.

	a (Å)	b (Å)	c (Å)	*η* = c/2a	x (Se)	*α* = *β* = *γ*
THM crystals	5.787	5.787	11.574	1	0.22989	90°
VBM crystals	5.7823	5.7823	11.6192	1.0047	0.22126	90°

### 3.5 The crystal structure of $CuInSe_{2}$

**Figure 3:**
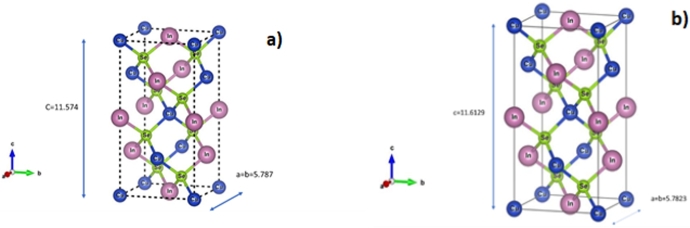
Schematic representation of *CuInSe*_2_ crystal structure conventional unit cell of height c, with a square base of width a for a) THM and b) VBM.

The final Rietveld refinement parameters used in the standardization of crystal structure and fractional coordinates modeled by the VESTA (Visualization for Electronics and Structural Analysis) program [23], as shown in Fig. 3. The crystal structure of $CuInSe_{2}$ well-established to be chalcopyrite corresponding to the space group I42d. $CuInSe_{2}$ well-characterized by tetrahedral coordination of every lattice site to its nearest neighbors. The very different chemical nature of the copper and indium atoms result in bonds between each of them and their neighboring selenium atoms with very different ionic character and lengths [24]. This bond-length has a very tiny change from $CuInSe_{2}$ crystal prepared by THM to VBM as listed on the following schematic representation, as illustrated in Fig. 3.

### 3.6 Grain size and micro-strain

The primary method in the X-ray diffraction is the line profile analysis based on diffraction line broadening. Diffraction line broadening can have many sources: size, strain, coherent precipitates, the mismatch between phases with different chemical compositions, etc. These effects can appear simultaneously, and their separation can sometimes be painful. Crystalline size effect and lattice distortions usually occur together. The procedure of separation complicated by the fact that strain broadening itself is complex as many different crystal defects cause it. In the following, the most essential and well-known techniques summarized.

### 3.7 Scherrer equation

Scherrer [25] attributed the full width to the Crystallite size effect obtained by the following equation:

(4) $$<L{>}_{vol}=\frac{K\lambda }{\Delta (2\theta )cos\theta }$$

where

(5) $$\Delta (2\theta )={\left[\Delta {\left(2\theta \right)}_{exp}^{2}-\Delta \theta_{I}^{2}\right]}^{\frac{1}{2}},$$

$\Delta {\left(2\theta \right)}_{exp}$ is the FWHM of the practical peak, $\Delta {\left(\theta \right)}_{I}=0.1$ is the broadening instrument, and $<L{>}_{vol}$ is the volume-weighted column length mean grain size. The Scherrer constant K is 0.9 for spherical particles. The grain size of the two crystals was 165 and 182 nm for the VBM and THM, respectively.

### 3.8 Classical Williamson-Hall plot

The Williamson–Hall method takes into account two effects, strain and size. This method gives an estimated value of volume–weight grain size $<D{>}_{vol}$, as well as the main square strain $<\epsilon^{2}>$. The classical Williamson–Hall plot given as [26].

(6) $$\Delta k=0.94/D_{vol}+2A\epsilon k$$

**Figure 4:**
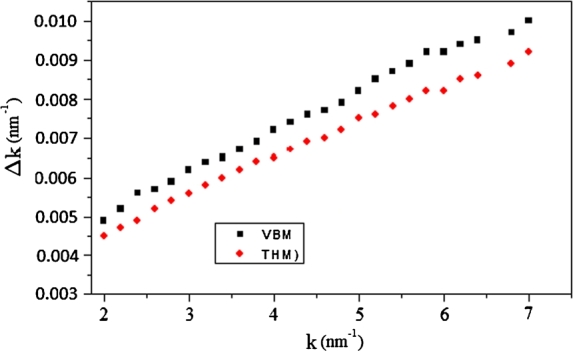
The relationship between Δk and k for VBM and THM crystals, according to the classical Williamson-Hall equation.

Where k = 2sin⁡θ/λ, Δk = cos⁡θ[Δ(2θ)]/*λ*, $<D{>}_{vol}$ is the average volume grain size and *ϵ* is the microstrain. Fig. 4 illustrates the relation between Dk and k of the two grown $CuInSe_{2}$ specimens according to the classical Williamson and Hall plot. From the intercept of the fitted straight line at k = 0 and the incline of the straight line, the average volume grain size, $<D{>}_{vol}$, and microstrain, *ϵ*, can be determined only through the constant A = 0.1 [27]. The values of $<D{>}_{vol}$ and *ϵ*, of the two crystals, are recorded in Table 1.

### 3.9 Warren–Averbach methods

The real part of the Fourier series coefficients A(L) given as purely size-broadening (S) and purely distortion broadening term (D) [28]:

(7) $$A(L)=A_{L}^{S}A_{L}^{D}$$

The distortion broadening coefficients can be written as [29]:

(8) $$\ln A_{L}^{D}=-2\pi^{2}L^{2}k^{2}<\epsilon_{g}^{2}>$$

**Figure 5:**
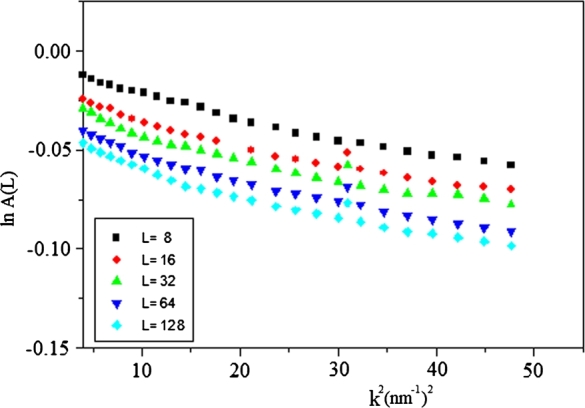
The relation between lnA_*L*_ and *k*^2^ for VBM crystal, according to classical Warren-Averbach equation.

The essential relation of the Warren–Averbach evaluation, then written as:

(9) $$\ln A(L)=\ln A_{L}^{S}-2\pi^{2}L^{2}k^{2}<\epsilon_{g}^{2}>$$

where L is the Fourier length obtained from $L=na_{3}$ [30], $a_{3}$ is the cell edge length in the parallel direction and expressed as:

(10) $$a_{3}=\frac{\lambda }{2(sin\theta_{2}-sin\theta_{1})}$$

n are integers starting from zero and $\left(\theta_{2}-\theta_{1}\right)$ is the angular range of the obtained diffraction side view. Fig. 5 illustrates the relationship between ln⁡A(L) and $k^{2}$ at various values of L, corresponding to the classical Warren–Averbach equation. For every L value, the data points must follow a straight line, the incline, and the intercept of these successive fixations at $k^{2}=0$ giving - 2$\pi^{2}L^{2}<\epsilon^{2}>$ and $\ln A_{L}$, respectively.

The size coefficient A(L) plotted as a function of L for the two crystals is illustrated in Fig. 7. The area-weighted mean length $<L{>}_{area}$ obtained by the tangent of small L value of A(L) extrapolated to the x-axis. However, the volume-weighted column length $<L{>}_{vol}$ obtained from the area under the curve of A(L) against L. The obtained value of $<L{>}_{area}$ and $<L{>}_{vol}$.= for the two crystals also listed in Table 1. The primarily obtained average column length of an ensemble of particles can be transformed into an average grain size of all the crystallites in the sample and have the same shape. The area-weighted average grain size $<D{>}_{area}$ and the volume-weighted grain size, $<D{>}_{vol}$, can be given by [20], [30], [31]

(11) $$<D{>}_{area}=\frac{3}{2}<L{>}_{area}$$

and

(12) $$<D{>}_{vol}=\frac{4}{3}<L{>}_{vol}$$

**Figure 6:**
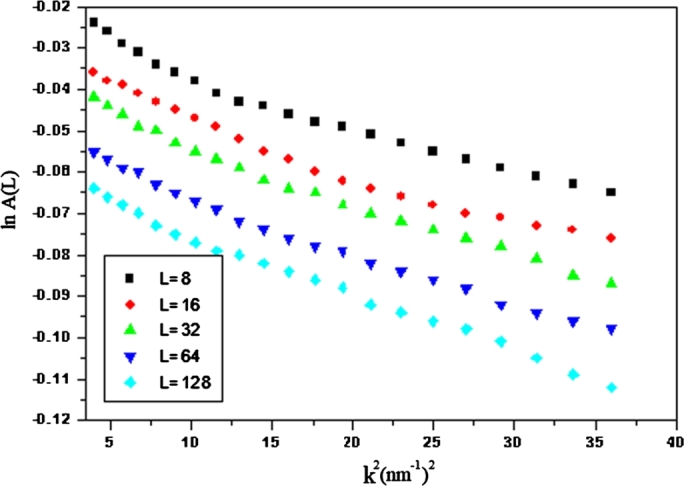
The relation between lnA_*L*_ and *k*^2^ for THM crystal, according to classical Warren-Averbach equation.

The values of $<D{>}_{area}$ and $<L{>}_{vol}$ were investigated and are listed in Table 1. The detailed evaluation of the Sharrer, Williamson–Hall, and Warren–Averbach equations described above applied for the two crystals listed in Table 1. From Table 1, the Scherrer method overestimates the volume-weighted column length, $<D{>}_{vol}$, likely because it did not split the broadening in the lattice from that due to the refined structure [32]. In both Williamson–Hall and Warren–Averbach methods, the microstructure parameters of the THM crystal are higher than those obtained from the VBM crystal, see Figs. 5 and 6. There are many different problems that cause strain — such as dislocation, stacking faults, unrelaxed mismatches between coherent phases, and severely distorted grain boundaries in nanocrystalline materials. In our samples, we consider the strain caused by dislocation.

**Figure 7:**
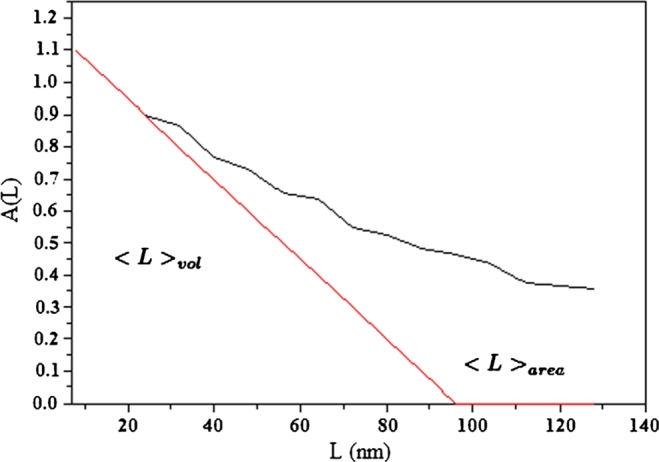
The relation between A(L) and L for THM crystals.

## 4 Conclusions

The effect of the growth technique on the structural properties of $CuInSe_{2}$ crystals was studied. The lattice constant of the VBM was higher than that of the THM. The Hot Probe Method results indicate that the crystal grown by the THM was a p-type and that grown by the VBM was an n-type. Three methods from X-ray line broadening determined the grain size and microstrain. The grain size of the crystal grown by the VBM was more significant than that grown by the THM. The microstrain of the grown crystals caused by dislocation.

## Declarations

### Author contribution statement

M. Mobarak, M.A. Zied: Conceived and designed the experiments; Performed the experiments.

M. Mostafa, M. Ashari: Analyzed and interpreted the data; Wrote the paper.

M. Mobarak and M.A. Zied have conceived, designed and performed the experiments.

M. Mostafa and M. Ashari have analyzed and interpreted the data and wrote the paper.

### Funding statement

This research did not receive any specific grant from funding agencies in the public, commercial, or not-for-profit sectors.

### Competing interest statement

The authors declare no conflict of interest.

### Additional information

No additional information is available for this paper.
